# Comparing the use of patient-reported outcomes in clinical studies in Europe in 2008 and 2018: a literature review

**DOI:** 10.1007/s11136-021-02946-7

**Published:** 2021-08-04

**Authors:** Guro Lindviksmoen Astrup, Gudrun Rohde, Stein Arne Rimehaug, Marit Helen Andersen, Tomm Bernklev, Kristin Bjordal, Ragnhild Sørum Falk, Nina Marie Høyning Jørgensen, Knut Stavem, Anita Tollisen, Cecilie Delphin Amdal, on behalf of PROMiNET

**Affiliations:** 1 Research support services, Oslo University Hospital, Oslo, Norway; 2 Department of Clinical Research, Sørlandet Hospital, Kristiansand, Norway; 3 Faculty of Health and Sport Sciences, University of Agder, Kristiansand, Norway; 4 Sunnaas Rehabilitation Hospital, Nesoddtangen, Norway; 5 Department of Transplantation Medicine, Oslo University Hospital, Oslo, Norway; 6 Faculty of Medicine, University of Oslo, Oslo, Norway; 7 Department of Research and Innovation, Vestfold Hospital, Tønsberg, Norway; 8 Medical Library at Ullevål Hospital, University of Oslo Library, Oslo, Norway; 9 Department of Pulmonary Medicine, Akershus University Hospital, Lørenskog, Norway; 10 Health Services Research Unit, Akershus University Hospital, Lørenskog, Norway; 11 Unger-Vetlesens Institute, Lovisenberg Diaconal Hospital, Oslo, Norway

**Keywords:** Patient-reported outcome, Patient-reported outcome measure, CONSORT-PRO, Review, Clinical studies

## Abstract

**Purpose:**

Several guidelines for the use of patient-reported outcomes (PROs) in clinical studies have been published in the past decade. This review primarily aimed to compare the number and compliance with selected PRO-specific criteria for reporting of clinical studies in Europe using PROs published in 2008 and 2018. Secondarily, to describe the study designs, PRO instruments used, patient groups studied, and countries where the clinical studies were conducted.

**Methods:**

A literature search was conducted in MEDLINE to identify eligible publications. To assess the number of publications, all abstracts were screened for eligibility by pairs of reviewers. Compliance with PRO-specific criteria and other key characteristics was assessed in a random sample of 150 eligible full-text publications from each year. Randomized controlled trials (RCTs) were assessed according to the full CONSORT-PRO checklist.

**Results:**

The search identified 1692 publications in 2008 and 4290 in 2018. After screening of abstracts, 1240 from 2008 and 2869 from 2018 were clinical studies using PROs. By full-text review, the proportion of studies discussing PRO-specific limitations and implications was higher in 2018 than in 2008, but there were no differences in the other selected PRO-specific criteria. In 2018, a higher proportion of studies were longitudinal/cohort studies, included ≥ 300 patients, and used electronic administration of PRO than in 2008. The most common patient groups studied were those with cancer or diseases of the musculoskeletal system or connective tissue.

**Conclusion:**

The number of clinical studies from Europe using PROs was higher in 2018 than in 2008, but there was little difference in compliance with the PRO-specific criteria. The studies varied in terms of study design and PRO instruments used in both publication years.

**Supplementary Information:**

The online version contains supplementary material available at 10.1007/s11136-021-02946-7.

## Introduction

A patient-reported outcome (PRO) is any report coming directly from patients, without interpretation by physicians or others, about how they function or feel in relation to a health condition and its therapy. PRO measures (PROMs) are instruments that obtain these patient reports [1]. PROMs capture issues important to patients, such as health-related quality of life (HRQoL), symptoms, or coping. These aspects are distinct from traditional endpoints such as survival, biological response, or observer-rated toxicity because they directly reflect the impact of disease and its treatment from the patient’s perspective [2].

A number of guidelines for the use of PROs have been developed over the last decade. These include minimum standards for use of PROs in clinical research (International Society for Quality of Life Research [ISOQOL]) [3], analyzing and reporting PRO results (Setting International Standards in Analyzing Patient-Reported Outcomes and Quality of Life Endpoints Data [SISAQOL]) [4], Consolidated Standards of Reporting Trials (CONSORT)-PRO [5]), and how to include PROs in protocols (Standard Protocol Items: Recommendations for Interventional Trials [SPIRIT-PRO]) [6] and in drug development [7]. In 2017, a preliminary report described the initial uptake of CONSORT-PRO from publication in 2013 to 2015 as high, with an increasing number of randomized controlled trials (RCTs) citing these guidelines [8]. Whether CONSORT-PRO has continued to contribute to an improvement in the use and quality of PROs in clinical research, including non-randomized studies, remain to be shown.

PROs can measure both the benefits and side effects of the treatment. Consequently, they have the potential to facilitate patient involvement in treatment decision-making and discussions of what the patient is willing to tolerate [2]. The use of PROs is particularly relevant to support treatment decisions in trials demonstrating a small or no difference in survival [9] and to support health policy decisions, including prioritization and organization of health services. PROs are also important in the evaluation of treatments and care for elderly and patients with chronic diseases, who emphasize the maintenance of quality of life and good function [10].

PROs were included in 27% of trials registered in ClinicalTrials.gov in 2007–2013 [11] and 45% in the Australian New Zealand Clinical Trials Registry (ANZCTR) from 2005 to 2017 [12]. However, most studies included PROs as secondary endpoints. Failure to report PROs may lead to under- or over-estimation of the effect of treatment [9, 13–15]. Many studies using PROs have insufficient quality, for example using PROMs with limited psychometric properties. Also, studies that are poorly reported, for instance failing to explain how the PROMs were administered, using non-representative samples, or lacking information on how missing data were handled, can leave the reader in doubt of the quality of the data [9, 13–21].

The primary aim of this review was to compare the number and compliance with PRO-specific criteria of published clinical studies conducted in Europe using PROs in 2008 versus 2018. Secondary aims were to describe the study designs, sample sizes, PROMs used, patient groups studied, and countries where the studies were conducted for each of the two years. We hypothesized that (1) the inclusion of PROs in clinical studies in Europe was higher in 2018 than in 2008 and that (2) a higher proportion of studies (absolute increase of at least 15%) complied with the selected PRO-specific criteria in 2018 compared to 2008.

## Methods

### Literature search

An experienced health care librarian (NMHJ) conducted a literature search for publications describing clinical studies using PROs. Clinical studies were defined as longitudinal/cohort and cross-sectional studies in addition to clinical trials. She searched MEDLINE using MeSH terms and text word variants for the concepts *patient reported outcome measures*, *quality of life* and *patients*. To limit work load, we chose a defined geographical area, and studies conducted in Europe were believed to be sufficient to answer the research questions. The search was then restricted to studies published in the English language in two different years, ten years apart. The last completed year (2018) prior to the literature search was selected and compared with 2008, which was before most of the guidelines were published. Case reports and review articles were excluded. The full search strategy is listed in Supplement 1.

### Screening of abstracts to identify eligible publications (part I)

Eleven PRO researchers participated in the review process. First, we pilot tested the literature search strategy and screening of abstracts in a sample of 500 publications. Approximately 2/3 of the publications were eligible for inclusion, and a full search was performed. All abstracts were independently reviewed for inclusion by two reviewers. Where discrepancy existed, this was resolved through discussion between the two and reviewed by a third author if needed.

The eligibility criteria were clinical studies published in 2008 or 2018 with patients from at least one European country and with a PRO or PROM mentioned in the title or abstract. Conference abstracts, editorials, opinion articles, scientific statements, guidelines, reviews, and non-English publications were deemed ineligible for inclusion. The main reason for ineligibility was documented for each paper. If there were more than one reason, we chose one reason in the following order: not clinical study including patients, no use of PROM, and non-European patients. We used EndNote software to keep track of the studies identified before exporting the library to Rayyan QCRI [22], a web application to administer literature reviews.

### Review of a subsample of full-text publications (part II)

The methodological aspects and compliance with PRO-specific criteria of the studies were evaluated through review of full-text publications in a random sample of studies from 2008 to 2018. A priori power calculation was performed where we assumed a 5% significance level and a power of 80%. To detect an absolute increase of at least 15% in the proportion of publications complying with selected CONSORT-PRO criteria, we had to review 150 full-text publications from each year. Thus, a random sample of 150 publications from part I for each year was selected using an algorithm from http://www.expertsearching.wordpress.com to extract the random publications from EndNote for evaluation in part II of the review. Pairs of two reviewers independently reviewed 60 full-text publications each. Where discrepancy existed, this was resolved through discussion between the two and reviewed by a third author if needed.

To compensate for publications that did not meet the eligibility criteria after full-text review, a new random selection was performed to reach the number of 150 full-text reviews for each year. To evaluate the representativeness of the random sample, we compared the percentage of publications categorized as an RCT in Rayyan QCRI, based on “key words” in the abstract, in part I (excluded those who were selected for part II) with the percentage in the random sample in part II.

To evaluate the methodological rigor of the studies independent of study design, we used five PRO-specific criteria obtained from the CONSORT-PRO extension published in 2013 [5]. After considering other criteria developed for evaluating PRO research, they were deemed most relevant also for studies using other designs and sufficient to evaluate difference in reporting of studies using PROs between the two publication years. These are

1. The PRO is identified in the abstract as a primary or secondary outcome;
2. The PRO hypothesis should be stated and relevant domains identified, if applicable;
3. Evidence of PROM validity and reliability should be provided or cited, if available;
4. Statistical approaches for dealing with missing data are explicitly stated for PROs pre-specified as primary or important secondary outcomes;
5. PRO-specific limitations and implications for generalizability of study findings and clinical practice are discussed.

The full CONSORT-PRO checklist intended for clinical trials [23] was used to evaluate reporting of the RCTs included. Several criteria were not relevant to all RCTs, such as reporting changes to trial outcomes after commencement (3b), interim analyses and stopping guidelines (7b), similarity of interventions (11b), additional analyses (12b), and why the trial ended (14b). When not relevant, the publications were scored as if they complied with the criteria to avoid negative results for the trials in question.

The results were stratified by design (RCT and non-RCT). We used data extraction forms to register key characteristics of the studies: publication year; study design (RCT, longitudinal/cohort, or cross-sectional); whether the study was single/multicenter; number of patients included; type (generic, disease-specific, or both) and name of PROM used; how the PROM was administered (electronic, in the clinic, postal, in this order if more than one mode of administration); whether compliance and user involvement were described; patient group (according to the World Health Organization’s International Classification of Diseases, 10th edition [ICD-10]); and country of patient recruitment. The PRISMA (Preferred Reporting Items for Systematic Reviews and Meta-Analyses Statements) [24] flow diagram was used to present the search results.

### Statistical analysis

Descriptive statistics are presented using number and percent. Comparison of proportions was performed by *χ*^2^ test or Fisher’s mid-p test, as appropriate. A difference was considered statistically significant when *p* < 0.05.

## Results

### Number of eligible publications (part I)

**Fig. 1:**
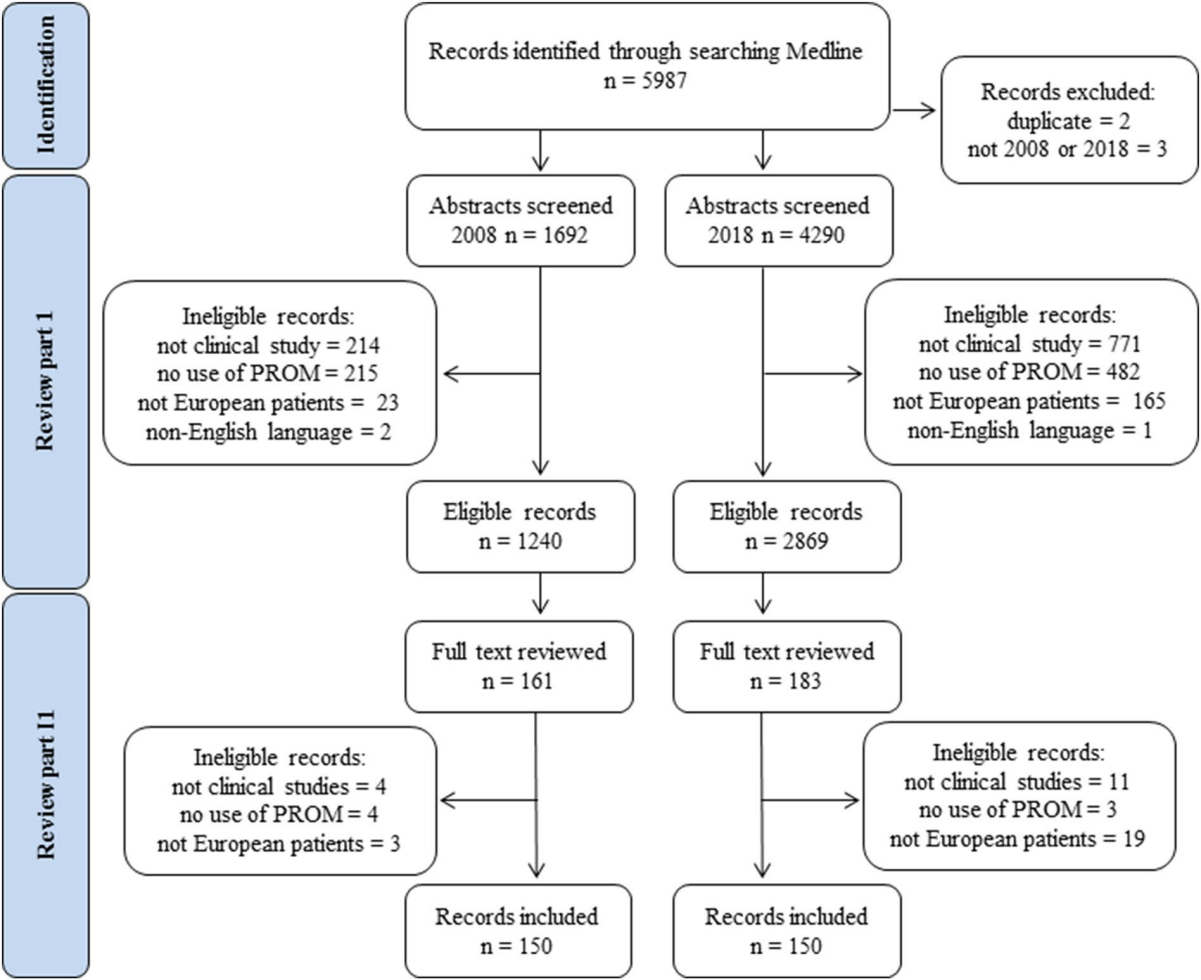
Flow chart of eligibility screening and inclusion Note: not clinical study = review article, protocol, case report, methodological development; no use of PROM = qualitative study, use of patient-reported experience measure

The literature search identified 5987 publications. Through the abstract screening, 31% of the publications were ineligible: 27% in 2008 and 33% in 2018 (Fig. 1). The most common reason for ineligibility in part I was that the publication did not report results from a clinical study with patients (e.g., review article, description of study protocol, case report, or study involving methodological development, such as validation of PROM). Of the eligible articles, 28% were published in 2008 and 72% in 2018.

### Compliance with PRO-specific criteria and PRO methodology (part II)

The random sample was found to be representative, i.e., the proportion of publications categorized as RCTs was similar in part I (12%) and in the random sample in part II (10%) (*p* = 0.27). Compliance with the full CONSORT-PRO checklist for RCTs (*n* = 44) is presented in Table 1 (difference in proportions was not tested statistically due to the limited number of RCTs). Two publications, both from 2018, met all criteria. For 18 of the 37 criteria, there was an absolute increase in compliance with CONSORT-PRO criteria of at least 15% in 2018 compared to 2008: for instance, description of sample size calculations (criterion 7a), numbers analyzed for PRO results (criterion 16), and trial limitations and implications (criterion 20). The largest difference was for criteria 23 and 24: registration of trial registry and access to trial protocol. Slightly more than half of the publications were the first (main) paper released from the RCTs in question (54% in 2008, 58% in 2018). Among publications where year of starting data collection was reported, the data collection started 4–9 years prior to publication in 2008 and 3–14 years prior to publication in 2018.

**Table 1:** Comparison of use of all CONSORT-PRO criteria between randomized controlled trials with PROMs published in 2008 and 2018, number of studies (%)

Item	Section/topic	2008 (*n* = 20)	2018 (*n* = 24)
	Title and abstract		
1a	Identification as a randomized trial in the title	10 (50)	15 (63)
1b	Structured summary (*P1b* PRO as primary/secondary outcome in abstract)	18 (90)	19 (79)
	Introduction		
2a	Scientific background and explanation of rationale	18 (90)	19 (79)
2b	Specific objectives or hypotheses (*P2b* PRO hypotheses and relevant domains stated)	13 (65)	13 (54)
	Methods		
3a	Description of trial design	17 (85)	23 (96)
3b	Important changes to methods after trial commencement, with reasons	**11 (55)**	**21 (88)**
4a	Eligibility criteria for participants	18 (90)	22 (92)
4b	Settings and locations where the data were collected	15 (75)	19 (79)
5	Interventions for each group with sufficient details to allow replication	**14 (70)**	**22 (92)**
6a	Completely defined pre-specified primary and secondary outcome measures (*P6a* Evidence of PRO instrument validity and reliability; methods of data collection)	12 (60)	12 (50)
6b	Any changes to trial outcomes after the trial commenced, with reasons	**10 (50)**	**19 (79)**
7a	How sample size was determined	**8 (40)**	**16 (67)**
7b	When applicable, explanation of any interim analyses and stopping guidelines	**11 (55)**	**19 (79)**
	Randomization		
8a	Methods used to generate the random allocation sequence	**8 (40)**	**16 (67)**
8b	Type of randomization; details of any restriction	**6 (30)**	**13 (54)**
9	Mechanism used to implement the random allocation concealment	**5 (25)**	**12 (50)**
10	Who: generated the random allocation sequence; enrolled participants; assigned participants to interventions	**6 (30)**	**13 (54)**
11a	If done, who was blinded after assignment to interventions and how	**11 (55)**	**20 (83)**
11b	If relevant, description of the similarity of interventions	**11 (55)**	**18 (75)**
12a	Statistical methods used to compare groups for primary and secondary outcomes (*P12a* Statistical approaches for dealing with missing data are explicitly stated)	13 (65)	13 (54)
12b	Methods for additional analyses	**14 (70)**	**21 (88)**
	Results		
13a	For each group, the numbers of participants who were randomly assigned, received intended treatment, and were analyzed for the primary outcome	13 (65)	18 (75)
13b	For each group, losses and exclusions after randomizations, with reasons	15 (75)	15 (63)
14a	Dates defining the periods of recruitment and follow-up	11 (55)	13 (54)
14b	Why the trial ended or was stopped	**13 (65)**	**20 (83)**
15	A table showing baseline demographic and clinical characteristics for each group	13 (65)	19 (79)
16	For each group, numbers of participants in each analysis	**10 (50)**	**18 (75)**
17a	For each primary and secondary outcome, results for each group, and the estimated effect size and its precision	15 (75)	15 (63)
17b	For binary outcomes, presentation of both absolute and relative effects sizes	**12 (60)**	**18 (75)**
18	Results of any additional analyses performed	15 (75)	21 (88)
19	All important harms or unintended effects in each group	12 (60)	14 (58)
	Discussion		
20	Trial limitations, addressing sources of potential bias, imprecision, and if relevant, multiplicity of analyses	**12 (60)**	**18 (75)**
21	Generalizability of the trial findings (*P20/21* PRO-specific limitations and implications for generalizability and clinical practice)	14 (70)	20 (83)
22	Interpretation consistent with results, balancing benefits and harms, and considering other relevant evidence	17 (85)	19 (79)
	Other information		
23	Registration number and name of trial registry	**2 (10)**	**17 (71)**
24	Where the full protocol can be assessed, if available	**2 (10)**	**11 (46)**
25	Sources of funding and other support, role of funders	16 (80)	19 (79)

*CONSORT-PRO* consolidated standards of reporting trials, *PRO* patient-reported outcome, *PROMs* patient-reported outcome measures

Differences in proportions between 2008 and 2018 ≥ 15% are highlighted in bold. PRO-specific extensions are prefaced by the letter *P*

Compliance with the five PRO-specific criteria for reporting for all studies (*n* = 300) is presented in Table 2. The most common criterion met in both 2008 and 2018 was *PRO identified in abstract as primary or secondary outcome*, while only a few studies reported on *statistical approaches for dealing with missing data*. The proportion of studies that discussed *PRO-specific limitations and implications for generalizability of study findings and clinical practice* was lower in 2008 than in 2018. Few studies met all five criteria, and there was no significant difference between 2008 and 2018.

**Table 2:** Comparison of use of PRO-specific criteria for reporting between studies with PROMs published in 2008 and 2018, number of studies (%)

PRO criteria	2008 (*n* = 150)	2018 (*n* = 150)	*p*-value
PRO identified as outcome in abstract	146 (97)	150 (100)	0.06*
PRO hypothesis stated	56 (37)	54 (36)	0.81
PROM validity and reliability cited	99 (66)	114 (76)	0.06
Statistical approaches for missing data stated	41 (27)	43 (29)	0.80
Discussed PRO-specific limitations and implications	107 (71)	138 (92)	** < 0.001**
Met all five criteria	10 (7)	18 (12)	0.11

*PRO* patient-reported outcome, *PROMs* patient-reported outcome measures

Statistically significant *p*-values are highlighted in bold. Tests performed using Fisher’s mid-p test are marked with an *

The proportion of RCTs was approximately the same in the reviewed samples from 2008 and 2018 (Table 3), while the proportion of longitudinal and cohort studies was lower in 2008 (31%) than in 2018 (49%). There were fewer large studies (≥ 300 participants) in 2008 than in 2018, but the proportion of multicenter studies was similar. Moreover, there was no difference in the proportions of generic or disease-specific PROMs used in 2008 and 2018. When reported, the most common mode of administration was in the clinic. From 2008 to 2018, there was a significant shift from postal to electronic administration. About 70% of the studies reported on compliance. Only one study, published in 2018, mentioned the inclusion of a user representative.

**Table 3:** Comparison of study design, type and mode of administration of PROM between studies published in 2008 and 2018, number of studies (%)

	2008 (*n* = 150)	2018 (*n* = 150)	*p*-value
Study design			
Randomized controlled trial	20 (13)	24 (16)	0.51
Cross-sectional study	81 (54)	50 (33)	** < 0.001**
Longitudinal/cohort study	49 (33)	76 (51)	**0.002**
Multicenter study	58 (39)	64 (43)	0.48
Number of patients included			
≤ 99	67 (45)	67 (45)	1.00
100–299	55 (37)	39 (26)	**0.04**
≥ 300	28 (19)	44 (29)	**0.04**
Type of PROM			
Generic	43 (29)	30 (20)	0.07
Disease-specific	40 (27)	46 (31)	0.45
Both	67 (45)	74 (49)	0.49
Mode of administration			
Electronic	2 (1)	10 (7)	0.02*
In the clinic	76 (51)	71 (47)	0.49
Post	36 (24)	15 (10)	**0.001**
Unknown	36 (24)	54 (36)	**0.02**
Described compliance	107 (71)	110 (73)	0.70
Described user representation	0 (0)	1 (1)	0.50*

*PROM* patient-reported outcome measure

Statistically significant *p*-values are highlighted in bold. Tests performed using Fisher’s mid-p test are marked with an *

In total, 340 different PROMs were used in the studies (Supplement 2) plus 29 non-validated single items or questionnaires designed for the specific study. The most commonly used generic PROMs were different versions of the Short-Form (SF-6/8/12/20/36, including three with RAND-36) and EQ-5D (Table 4). The EQ-5D was more frequently used in 2018 than in 2008. The most commonly used disease-specific PROM was the European Organization for Research and Treatment of Cancer core questionnaire and modules. The Hospital Anxiety and Depression Scale was most often used for measuring anxiety or depression, while pain was most often measured by a visual analog scale.

**Table 4:** Distribution of the most frequently used (*n* > 5) PROMs by publication year, number of studies

Patient-reported outcome measure	2008	2018
Short form (SF)/RAND		
SF/RAND-6/8/12/20	5	11
SF/RAND-36	45	28
EQ-5D	15	36
European Organization for Research and Treatment of Cancer core (EORTC QLQ-C30) and/or module	23	25
Visual Analog Scale (VAS) for pain or other symptom	22	19
Hospital Anxiety and Depression Scale (HADS)	13	14
World Health Organization Quality of Life (WHOQOL)	8	4
Saint George’s Respiratory Questionnaire (SGRQ)	5	5
Beck Depression Inventory (BDI)	6	3
Symptom Checklist (SCL) 27/90/90r	8	0
Western Ontario and McMaster Universities Osteoarthritis Index (WOMAC)	4	2
Patient Health Questionnaire (PHQ)	2	4
Knee Injury and Osteoarthritis Outcome Score (KOOS)	0	7

*PROMs* patient-reported outcome measures

The numbers do not sum up to 150 in each year due to inclusion of several patient-reported outcome measures in each study

The two most common patient groups studied in both 2008 and 2018 were those with cancer and diseases of the musculoskeletal system or connective tissue (Table 5).

**Table 5:** Patient groups included in the studies by publication year, number of studies

Patient group	2008 (*n* = 150)	2018 (*n* = 150)
1. Infectious and parasite diseases	2	2
2. Cancer	30	26
3. Haematologic and immune diseases	2	2
4. Endocrine diseases	8	6
5. Mental illness	5	6
6. Diseases in the nervous system	17	14
7. Diseases in the eye	2	2
8. Diseases in the ear	1	3
9. Diseases in the circulatory system	11	16
10. Diseases in the respiratory system	11	14
11. Diseases in the digestive system	13	8
12. Skin diseases	10	3
13. Diseases in the musculoskeletal system and connective tissue	14	33
14. Diseases in the urinary and genital organs	12	7
15. Pregnancy, birth, postnatal period	0	0
16. Children	2	0
17. Other (injury etc.)	10	8

Patient groups are categorized according to the World Health Organization’s International Classification of Diseases, 10th edition (ICD-10)

The studies were conducted in 30 different European countries, although some multicenter studies also extended into other continents. The proportion of international studies was lower in 2008 (5%) than in 2018 (16%, *p* = 0.002) (Table 6). Patients from Germany, the United Kingdom, and the Netherlands were most often included. Of the studies with participants from non-European countries, patients from the USA (16 studies) and Canada (10 studies) were most often included.

**Table 6:** Distribution of countries where the studies were conducted by publication year, number of studies

Country	2008 (*n* = 150)	2018 (*n* = 150)
The Netherlands	25	22
Germany	24	29
The United Kingdom	20	32
Sweden	17	7
Italy	13	23
France	13	15
Switzerland	9	7
Austria	5	5
Spain	4	22
Belgium	4	9
Norway	4	8
Denmark	4	6
Poland	3	6
The Czech republic	1	5
Bosnia–Herzegovina, Bulgaria, Croatia, Estonia, Finland, Hungary, Greece, Iceland, Ireland, Macedonia, Portugal, Romania, Russia, Serbia, Slovakia, Ukraine	< 5	< 5
Involving more than one country	7	24

The number of studies in each country does not sum up to 150 in each year due to multicenter studies with contribution from several countries

## Discussion

The main finding in this review was that the overall number of publications with PROs was higher in 2018 than in 2008. This may indicate an increasing interest in including patients’ perspectives in clinical research, which can facilitate patient involvement in treatment decision-making and provide guidance for health-care decisions [2]. This finding supports previous reviews reporting increased numbers of clinical trials with PROs in ClinicalTrials.gov (2007–2013) [11] and ANZCTR (2005–2017) [12]. In the present study, a higher proportion of the identified publications from 2018 were ineligible for inclusion than in 2008. A higher proportion of the studies were “non-clinical studies,” e.g., protocols or methodological studies, and “not using PROM.” This may be due to more focus on assessment of validity and reliability of PROMs and more studies using qualitative research or patient-reported experience measures (PREMs) in 2018 than in 2008. More studies in the sample from 2018 included non-European patients, which may reflect an increase in the number of studies using PROs outside this region.

It is notable that only two RCTs, both published in 2018, complied with all CONSORT-PRO criteria [25, 26]. Several criteria had a high compliance in both years such as 4a (i.e., eligibility criteria for participants), while some had a low compliance in both years, such as 14a (i.e., dates defining the periods of recruitment and follow-up). This may indicate that release of the CONSORT-PRO has had limited impact on reporting so far, which was also found in an earlier review on the topic [19]. The reason for this is not clear, but worth noting; our review revealed some uncertainty or disagreement about the interpretation of the CONSORT-PRO criteria among the reviewers. Perhaps clinical researchers may perceive the CONSORT-PRO as too ambiguous or comprehensive and therefore fail to use it. It is worth noting that almost half of the publications were not the first (main) publication from the RCTs in question, and more information may have been published elsewhere. In addition, data collection started 3–14 years prior to publication in 2018, meaning that some studies were planned prior to the release of the latest version of the CONSORT-PRO in 2010, and this may have impacted the possibility to meet all criteria.

The proportion of all studies complying with the selected five PRO-specific criteria for reporting in 2008 and 2018 differed for only one criterion. Studies citing the CONSORT-PRO were associated with improved PRO reporting the first years after publication of the CONSORT-PRO extension [8]. However, many studies may have been planned or conducted prior to the release of the CONSORT-PRO in 2013, up to 14 years prior to publication in one study. Still, the concepts described have been central in PRO research for many years. Other guidelines for PRO research [3, 4, 6, 7] are also relatively new and may not have reached their full impact yet.

Almost all studies identified a PRO as outcome in the abstract irrespective of publication year. This is consistent with a previous review of RCTs in oncology where 81% identified a PRO [17] and not surprising given the search terms used in the literature search. A PRO hypothesis, a criterion that only applies for RCTs (15% of the studies in this review), was stated in slightly more than half of the RCTs. This might be due to PROs being secondary aims or explorative endpoints of many studies [17]. Still, stating a PRO hypothesis should be encouraged. Failure to report a pre-specified PRO hypothesis weakens study results as the reader may be in doubt of whether there is selective reporting or multiple testing [6].

About 3/4 of all studies published in 2018 reported or cited evidence of PROM validity and reliability, while only about half of the RCTs completely defined pre-specified primary and secondary outcome measures (including how and when they were assessed). The proportion of studies reporting or citing evidence of PROM validity and reliability was similar to that reported for RCTs in oncology [17], which may suggest that researchers regard validity and reliability as important regardless of study design. Having valid and reliable PROMs is a prerequisite to ensure robust study results that can be used in clinical practice [5]. To ensure the readers’ confidence in the results, such information should be made available.

The proportion of RCTs that stated statistical approaches for dealing with missing data was similar in 2008 and 2018, although for both years, higher than reported for RCTs in oncology [17]. Still, it is surprising that fewer than half of all studies reported this, as missing data lead to reduced power, is a potential source of bias and can result in misleading results [5].

Discussing PRO-specific limitations and implications for generalizability of study findings and clinical practice was more prevalent in 2018 than in 2008. This may reflect an increased understanding of and focus on methodological issues in PRO research for interpretation of data. It may also reflect that researchers want to improve patient treatment through the use of PROs in clinical research.

In 45% of the studies in both 2008 and 2018, fewer than 100 patients were included. Sample size estimates are usually based on the primary endpoint, which may or may not be a PRO. Few included patients may also be due to rare patient groups, small study centers, or difficulty in recruiting patients for logistical or other reasons. This is a concern, because small samples can lead to underpowered studies, without the possibility to answer the research question of interest [27], redundant research, and wasted resources. For rare patient groups and small centers, multicenter studies should be encouraged to increase the sample size and statistical power. However, there was no difference in the proportion of multicenter studies between 2008 and 2018.

The selected studies used many different PROMs. Some studies did not report a specific PROM, but used non-validated or ad hoc single items or questionnaires developed for their study. The use of non-validated questionnaires or single items from original questionnaires without validation is not recommended because of uncertainty whether the questionnaire measures what it is intended to [2]. Moreover, the findings from such measures may be difficult to compare with other studies. The most commonly used PROMs in this review have been rigorously tested for reliability and validity, such as the EQ-5D and the SF-36. A large proportion of the included studies used the EQ-5D instrument. This was originally developed for use in health economic analyses [28], but it is also used as a simple and short measure of HRQoL. The frequent use of EQ-5D in 2018 compared to 2008 may reflect an increase in the number of clinical registries, where this instrument often is included.

More than 70% of the studies included some description of compliance or dropout, such as the number of invited subjects and number who completed PROMs in cross-sectional studies, or dropouts during the study in RCTs and longitudinal/cohort studies. It is worrying that many studies failed to report mode of administration of PROM, as this may nurture many clinicians skepticism about PRO reliability [29].

Several studies used more than one mode of administration, which has different advantages and disadvantages. As expected, more studies used electronic PROMs in 2018 than in 2008. Many investigators prefer electronic administration, as this facilitates data entry and reduces missing data compared with paper and pencil. On the other hand, there may be accessibility issues that introduce selection bias with electronic administration, while patients may feel less comfortable disclosing sensitive topics in the clinic [29]. However, a meta-analysis reported that mode of administration does not seem to affect the patients’ response, i.e., increase bias and that the use of a mix of modes of administration may maximize response rates because different modes may be suitable for different patients or patient groups [30, 31].

Only one study explicitly reported on any type of user (or patient) representation. Many funders or ethical review committees now request documentation on user representation in applications and protocols, but it is not required to report such collaboration in publications. Many of the reviewed studies were planned and conducted several years prior to publication, when such user representation was less common.

### Strengths and limitations

This review has several strengths. It assessed the differences in the number and methodology of clinical studies using PROs in 2 years with ten years interval. Several important guidelines, such as the CONSORT-PRO criteria, were published during those 10 years and could have influenced the reporting of the publications. The review assessed diverse studies with different designs in different countries and in a wide range of patient groups. The findings could thus be used for comparison in the future.

Some limitations should be noted. The decision to use only the five PRO-specific criteria from the CONSORT-PRO extension and not the full checklist could be questioned. PRO-specific *elaborations* presented in the same publication could have been used in addition. Several other CONSORT-PRO criteria also apply to all study designs. However, the intention of this review was not to assess whether the studies complied with the full CONSORT-PRO checklist, but to evaluate the PRO methodology, and for this purpose, the five items were considered sufficient, together with evaluation of other key characteristics such as number of patients included, inclusion of user representatives, and type of PROM used. Several researchers with different backgrounds were involved in the review process. A number of publications included in part I of the review were excluded in part II after closer scrutiny. A more stringent preparation phase could have resulted in a more concise evaluation of eligibility in part I. Furthermore, the review revealed some uncertainty or disagreement about the interpretation and operationalization of the CONSORT-PRO criteria among the reviewers, and a pragmatic approach was chosen. For example, a study would be eligible if it reported on validity *or* reliability for one of the several PROMs included in the study, but not necessarily for the patient group in question or in the language of administration. Similarly, statistical approaches for dealing with missing data varied from the exclusion of respondents with missing data to the use of more advanced statistical analyses to accommodate missing data, such as linear mixed models. Due to this uncertainty, there were several clarifying discussions during the review process, which could have been exposed at an earlier stage or avoided if we had included pairwise review in the pilot study or in part I, or conducted a pilot test in part II. Finally, the review included studies conducted in European countries that may differ from those conducted in other parts of the world. In addition, publications by systematic search in only one database were included, and searches in other databases could have led to different results. Also, our random selection of 300 publications may not be entirely representative.

### Conclusion

The number of clinical studies using PROs in Europe was higher in 2018 than in 2008, and a few methodological aspects seemed to have improved. Altogether, there was little difference between 2008 and 2018 in compliance with the PRO-specific criteria for reporting. Therefore, it seems that published guidelines have had limited impact on the reporting of clinical studies using PROs so far. The large variations in the methodology and reporting of published PRO research may limit the use of PROs to reach its full potential in terms of influence. Higher influence may facilitate the use of PROs to support treatment decisions, health policy, and improve patient care.

## Supplementary Information

Below is the link to the electronic supplementary material.

Supplementary file1 (DOCX 16 kb)

Supplementary file2 (DOCX 19 kb)
