# Supplementary material for: Comparing the use of patient-reported outcomes in clinical studies in Europe in 2008 and 2018: a literature review
Source: Qual Life Res. 2021 Aug 4;31(3):659–69. doi: 10.1007/s11136-021-02946-7 (PMC8921066; doi:10.1007/s11136-021-02946-7)
Supplement: Supplementary file 1 — Supplementary file1 (DOCX 16 kb) [file 11136_2021_2946_MOESM1_ESM.docx]

Supplement 1

MEDLINE (Ovid) search strategies (Database: Ovid MEDLINE(R) and Epub Ahead of Print, In-Process & Other Non-Indexed Citations and Daily <1946 to October 19, 2018>)

2008

| 1. exp Patient Reported Outcome Measures/ |
| --- |
| 2. exp patient outcome assessment/ |
| 3. (patient reported adj3 (outcome* or measure* or assessment*)).tw,kf. |
| 4. (patient centered adj3 (outcome* or measure* or assessment*)).tw,kf. |
| 5. (self reported adj3 (outcome* or measure* or assessment*)).tw,kf. |
| 6. (patient oriented adj3 (outcome* or measure* or assessment*)).tw,kf. |
| 7. (proms or prom).tw,kf. |
| 8. or/1-7 |
| 9. exp "Quality of Life"/ |
| 10. (quality adj3 life).ti,kf. |
| 11. QoL.ti,kf. |
| 12. hrqol.ti,kf. |
| 13. hr-qol.ti,kf. |
| 14. or/9-13 |
| 15. 8 or 14 |
| 16. exp Patients/ |
| 17. (patients or inpatient* or in-patient*).tw,kf. |
| 18. (outpatient* or out-patient*).tw,kf. |
| 19. 16 or 17 or 18 |
| 20. 15 and 19 |
| 21. 2008*.yr,dp,so,dt. |
| 22. 20 and 21 |
| 23. limit 20 to yr="2008" |
| 24. 22 or 23 |
| 25. exp europe/ |
| 26. (Italy or Italian* or Italien* or Rome or Roma or Liechtenstein or Luxembourg* or Mediterranean or Monaco or Netherland* or Amsterdam or Flemish* or Flanders or Portugal or Portugue* or Lisboa or San Marino or Scandinavia* or Nordic Countr* or Northern countr* or Norway or Norweg* or Norge or Oslo or Denmark or Danish or Danes or Danmark or Copenhagen or Kobenhavn or Sweden or Swedish or Swedes or Sverige or Stockholm or Goteborg or Iceland* or Reykjavik or Finland or Finnish* or Tampere or Helsinki or Spain or Spanish or Espana or Barcelona or Madrid or Switzerland or Bern or Geneva or Transcaucasia or Armenia or Yerevan or Azerbaijan or Baku or Georgia or Tbilisi or Kazakhstan or Astana or Kyrgyzstan or Bishkek or Moldova or Chisinau or Ukraine or Kiew or Kiev or Uzbekistan or Tashkent or Vatican City or Albania or Tirana or Baltic States or Estonia or Tallinn or Latvia or Riga or Lithuania or Vilnius or Bosnia or Herzegovina or Sarajevo or Bulgaria or Sofia or Croatia or Zagreb or Czech or Prague or Hungary or Hungarian* or Budapest* or Kosovo or Pristina or Macedonia or Skopje or Moldova or Montenegro or Podgorica or Poland or Warszawa or Warsaw or Belarus or Minsk or Romania or Bucuresti or Russia or Moscow or Saint Petersburg or Bashkiria or Dagestan or Tatarstan or Serbia or Beograd or Slovakia or Bratislava or Slovenia or Ljubljana).tw,kf,ia,in. |
| 27. (Andorra or Austria* or Osterreich or Vienna or Wien or Balkan or Belgium or Belgian or Brussel* or France or French or Paris or Germany or German or Germans or Hamburg or Berlin or Deutschland or Gibraltar or United Kingdom or Channel Islands or England or London or Ireland or Great Britain or Irish or Dublin or Scotland or Scottish or Edinburg or Wales or Welsh or Cardiff or Greece or Greek* or Athens or (Bergen adj3 (universit* or hospital* or Center))).tw,kf,ia,in. |
| 28. 25 or 26 or 27 |
| 29. 24 and 28 |
| 30. limit 29 to english language |
| 31. limit 30 to "reviews (best balance of sensitivity and specificity)" |
| 32. limit 30 to (case reports or systematic reviews) |
| 33. 31 or 32 |
| 34. 30 not 33 |
| 35. (1998* or 1999* or 2000* or 2001* or 2002* or 2003* or 2004* or 2005* or 2006* or 2007* or 2009* or 2010* or 2011* or 2012* or 2013* or 2014* or 2015* or 2016* or 2017* or 2018* or 2019*).yr,dp,so. |
| 36. 34 not 35 |

2018

| 1. exp Patient Reported Outcome Measures/ |
| --- |
| 2. exp patient outcome assessment/ |
| 3. (patient reported adj3 (outcome* or measure* or assessment*)).tw,kf. |
| 4. (patient centered adj3 (outcome* or measure* or assessment*)).tw,kf. |
| 5. (self reported adj3 (outcome* or measure* or assessment*)).tw,kf. |
| 6. (patient oriented adj3 (outcome* or measure* or assessment*)).tw,kf. |
| 7. (proms or prom).tw,kf. |
| 8. or/1-7 |
| 9. exp "Quality of Life"/ |
| 10. (quality adj3 life).ti,kf. |
| 11. QoL.ti,kf. |
| 12. hrqol.ti,kf. |
| 13. hr-qol.ti,kf. |
| 14. or/9-13 |
| 15. 8 or 14 |
| 16. exp Patients/ |
| 17. (patients or inpatient* or in-patient*).tw,kf. |
| 18. (outpatient* or out-patient*).tw,kf. |
| 19. 16 or 17 or 18 |
| 20. 15 and 19 |
| 21. 2018*.yr,dp,so,dt. |
| 22. 20 and 21 |
| 23. exp europe/ |
| 24. (Italy or Italian* or Italien* or Rome or Roma or Liechtenstein or Luxembourg* or Mediterranean or Monaco or Netherland* or Amsterdam or Flemish* or Flanders or Portugal or Portugue* or Lisboa or San Marino or Scandinavia* or Nordic Countr* or Northern countr* or Norway or Norweg* or Norge or Oslo or Tromso or Trondheim or Denmark or Danish or Danes or Danmark or Copenhagen or Kobenhavn or Sweden or Swedish or Swedes or Sverige or Stockholm or Goteborg or Iceland* or Reykjavik or Finland or Finnish* or Tampere or Helsinki or Spain or Spanish or Espana or Barcelona or Madrid or Switzerland or Bern or Geneva or Transcaucasia or Armenia or Yerevan or Azerbaijan or Baku or Georgia or Tbilisi or Kazakhstan or Astana or Kyrgyzstan or Bishkek or Moldova or Chisinau or Ukraine or Kiew or Kiev or Uzbekistan or Tashkent or Vatican City or Albania or Tirana or Baltic States or Estonia or Tallinn or Latvia or Riga or Lithuania or Vilnius or Bosnia or Herzegovina or Sarajevo or Bulgaria or Sofia or Croatia or Zagreb or Czech or Prague or Hungary or Hungarian* or Budapest* or Kosovo or Pristina or Macedonia or Skopje or Moldova or Montenegro or Podgorica or Poland or Warszawa or Warsaw or Belarus or Minsk or Romania or Bucuresti or Russia or Moscow or Saint Petersburg or Bashkiria or Dagestan or Tatarstan or Serbia or Beograd or Slovakia or Bratislava or Slovenia or Ljubljana).tw,kf,ia,in. |
| 25. (Andorra or Austria* or Osterreich or Vienna or Wien or Balkan or Belgium or Belgian or Brussel* or France or French or Paris or Germany or German or Germans or Hamburg or Berlin or Deutschland or Gibraltar or United Kingdom or Channel Islands or England or London or Ireland or Great Britain or Irish or Dublin or Scotland or Scottish or Edinburg or Wales or Welsh or Cardiff or Greece or Greek* or Athens or (Bergen adj3 (universit* or hospital* or Center))).tw,kf,ia,in. |
| 26. 23 or 24 or 25 |
| 27. 22 and 26 |
| 28. (1998* or 1999* or 2000* or 2001* or 2002* or 2003* or 2004* or 2005* or 2006* or 2007* or 2008* 2009* or 2010* or 2011* or 2012* or 2013* or 2014* or 2015* or 2016* or 2017* or 2019*).yr,dp,so. |
| 29. 27 not 28 |
| 30. limit 29 to english language |
| 31. limit 30 to "reviews (best balance of sensitivity and specificity)" |
| 32. limit 30 to (case reports or meta analysis or systematic reviews) |
| 33. 31 or 32 |
| 34. 30 not 33 |
| 35. remove duplicates from 34 |
