# Supplementary material for: Comparing the use of patient-reported outcomes in clinical studies in Europe in 2008 and 2018: a literature review
Source: Qual Life Res. 2021 Aug 4;31(3):659–69. doi: 10.1007/s11136-021-02946-7 (PMC8921066; doi:10.1007/s11136-021-02946-7)
Supplement: Supplementary file 2 — Supplementary file2 (DOCX 19 kb) [file 11136_2021_2946_MOESM2_ESM.docx]

Supplement 2 Patient-reported outcome measures used in the studies (n=300)

| 15D | International Onychomycosis-specific Questionnaire |
| --- | --- |
| 2000 International Knee Documentation Committee | International Physical Activity Questionnaire |
| Acceptance of Illness Scale | International Prostate Symptom Score |
| Acne Quality of Life Ouestionnaire | International RLS Severity Scale |
| Activity of Daily Living scale | Interview Schedule for Social Interaction |
| ALS Functional Rating Scale | Inventory for Assessing Quality of Life in Children and Adolescents |
| Anal Sphincter-Conservative Treatment | Inventory for Social Support |
| Ankylosing Spondylitis Disease Activity Score | Itch Numeric Rating Scale |
| Anxiety Scale Questionnaire | ItchyQoL |
| Arthritis Impact Measurement Scales 2 Short Form | Kaasa Test |
| Asthma Quality of Life Questionnaire | Kansas City Cardiomyopathy Questionnaire |
| Atrial Fibrillation Effect on QualiTy-of-life | KIDSCREEN-52 |
| Bath Ankylosing Spondylitis Disease Activity Index | KINDL |
| Bath Ankylosing Spondylitis Functional Index | Kings health questionnaire |
| Bath Ankylosing Spondylitis Metrology Index | Knee Injury and Osteoarthritis Outcome Score |
| Bath Ankylosing Spondylitis Patient Global Score | Knee Society Score |
| Bech-Rafaelsen Mania Scale | Lancashire Quality of Life Profile |
| Bech-Rafaelsen Melancholia Scale | Lattinen Index |
| Beck Depression Inventory | Lawton Instrumental Activities of Daily Living |
| Beck Scale for Suicidal Ideation | Leeds Dyspepsia Questionnaire |
| Benefit Finding Scale | Leicestershire Urinary Symptoms Questionnaire |
| Body Concept Scale | Life Habits assessment - general short form |
| Body Image Scale | Life Quality Index |
| BODY-Q | Life Satisfaction Checklist |
| Boston Carpal Tunnel Questionnaire | Lifestyle Changes Questionnaire for Caregivers of Severe Acquired Brain Injury Patients |
| BREAST-Q | List of Threattening Life Events Questionnaire |
| Brief Pain Inventory | Lower Extremity Functional Score |
| Brief Symptom Inventory | Male Sexual Health Questionnaire |
| BriefCOPE | Marital Satisfaction Questionnaire for Older Persons |
| Brompton breathing pattern assessment tool | Massachusetts Sleep Diary |
| Camberwell Assessment of Need Short Appraisal Schedule | McGill Pain Questionnaire |
| Cancer Lineal Analog Scales | Meaning in Life Questionnaire |
| Cancer Therapy Satisfaction Questionnaire | Measurement of Urinary Handicap |
| Cantril's ladder | Medical Outcomes Study Sleep Scale index |
| Caregiver Stress Scale | Medical Research Council Breathlessness score |
| Carlsson and Dent reflux questionnaire | Middlesex Hospital Questionnaire |
| CECA10 | Migraine Disability Assessment Questionnaire |
| Centre for Epidemiologic Studies – Depression scale | Migraine Physical Function Impact Diary |
| Chalder Fatigue Scale | Mini International Neuropsychiatric Interview |
| Checklist Individual Strength | Mini Mental State Examination |
| Child and Adolescent Mindfullness Measure | Mini Mental Test |
| Child Behavior Checklist | Minnesota Living with Heart Failure Questionnaire |
| Child Health Questionnaire | MND Social Withdrawal Scale |
| Child Health Questionnaire - Parent Form 50 | Modified Scale of the Medical Research Council |
| Child Oral health Impact Profile | Moorehead-Ardelt Quality of Life Questionnaire |
| Chronic Resporatory Questionnaire | [Movement Disorder Society - Unified Parkinson's Disease Rating Scale](https://www.sciencedirect.com/topics/medicine-and-dentistry/stereotypic-movement-disorder) |
| Chronic Venous Insufficiency Questionnaire | MS Quality of Life 54 |
| Cincinnati Knee Rating Scale | Multidimensional Quality of Life Questionnaire for HIV/AIDS |
| Client Satisfaction Questionnaire-8 | Multidimentional Fatigue Inventory |
| Clinical Global Impression Severity Scale (CGI-S) | Multidimentional Health Locus of Control Questionnaire |
| Clinical Impairment Assessment | Nail Psoriasis Severity Index |
| Close Persons Questionnaire | National Eye Institute Visual Function Questionnaire 25 |
| Cognitive Failures Questionnaire | Neurogenic Bowl Dysfunction |
| Community Integration Questionnaire | Neuropathic Pain Symptom |
| Compliance Questionnaire for Rheumatology | Neuropsychiatric Inventory |
| Confidence in Diabetes Self-care scale | Nijmegen Questionnaire |
| Confusion Assessment Method | Nonarthritic Hip Score |
| COPD Assessment Test | Norfolk Quality of Life- Diabetic Neuropathy |
| COPD Author’s Questionnaire | Nottingham Health Profile |
| COPE Inventory | Ocular Surface Disease Questionnaire |
| Coping of Rheumatic Stressors questionnaire | Oldenburg Inventory Questionnaire |
| Coping Strategies Questionnaire | Olerud-Molander Ankle Scores |
| Coping with Health Injuries and Problems scale | Oral Health Impact Profile |
| Core Outcome Measures Index | Oswestry Disability Index |
| Crohn's and Ulcerative Colitis questionnaire | Over-active Bladder Questionnaire |
| CTCAE (patient vs doctor report) | Oviedo Sleep Questionnaire |
| Cushing QOL questionnaire | Oxford Hip Score |
| Cystic Fibrosis Questionnaire - revised | Oxford Knee Score |
| Davos Assessment of Cognitive Biases | Oxford Shoulder Score |
| Defecation Distress Inventory | Parkinson's Disease Questionnaire 39 |
| Demoralization Scale | Patient Acceptable Symptom State |
| Dermatology Life Quality Index | Patient Global Satisfaction assessment |
| Deterioration in Daily Living Activities in Dementia | Patient Health Questionnaire |
| Diabetes Family Behavior Scale | Patient Willingness to use drug again assessment |
| Diabetes Family Conflict Scale | PCV metra |
| Diabetes Quality of Life | Pearlin Mastery Scale |
| Diabetes Self-care Inventory | Pediatric Quality of Life Inventory |
| Diabetes Treatment Satisfaction Questionnaire | Pelvic Organ Prolapse/Urinary Incontinence Sexual Questionnaire |
| Diabetes-specific Health-Related Quality of Life | Perceived Stress Questionnaire |
| Disabilities of the Arm | Perception of Anticoagulant Treatment Questionnaire |
| Disabilities of the Arm, Shoulder and Hand Score | Physical Symptom Checklist |
| Disability Rating Index | Pittsburgh Sleep Quality Index |
| Dissociation Questionnaire | Positive and Negative Syndrome Scale |
| Dissociative Experiences Scale | Posttraumatic Growth Inventory |
| Ditrovie scale | Posttraumatic Stress Disorder Checklist |
| Dukes Activity Status Index | Preference of Medicine questionnaire |
| Dutch Heart Failure Knowledge | Problem Areas in Diabetes scale |
| Dyadic Adjustment Scale | Profile of Mood State |
| Dyspnea-12 Questionnaire | Prolapse Quality of Life questionnaire |
| Eating Assessment Tool-10 | Psoriatic Arthritis Disease Activity Score |
| Eating Disorder Examination-Questionnaire | Psoriatic Arthritis Quality of Life questionnaire |
| Eating Disorder Inventory | Psychological General Well-being Index |
| Edmonton Symptom Assessment System | Quality of Life in Epilepsy |
| Eosinophilic Esophagitis Quality of Life Adult | Quality of Life in RLS |
| Epworth Sleepiness Scale | Quality of Life Questionnaire |
| European Health Literacy Survey project questionnaire | Quality of Life Scale |
| European Heart Failure Self-Care Behaviour scale | Questions on Life Satisfaction Modules - Fragen zur Lebenszufriedenheit |
| European Organization for the Research and Treatment of Cancer Quality of Life Questionnaire-BR23 | Recent Life Event Questionnaire |
| European Organization for the Research and Treatment of Cancer Quality of Life Questionnaire-C15PAL | Reflux Symptom Index |
| European Organization for the Research and Treatment of Cancer Quality of Life Questionnaire-C30 | Rheumatoid Arthritis Impact of Disease score |
| European Organization for the Research and Treatment of Cancer Quality of Life Questionnaire-CX24 | Rhinosinusitis Outcome Measure |
| European Organization for the Research and Treatment of Cancer Quality of Life Questionnaire-F13 | Rosenberg’s Self-Esteem Scale |
| European Organization for the Research and Treatment of Cancer Quality of Life Questionnaire-H&N35 | Satisfaction With Health |
| European Organization for the Research and Treatment of Cancer Quality of Life Questionnaire-INFO25 | Satisfaction With Life Scale |
| European Organization for the Research and Treatment of Cancer Quality of Life Questionnaire-LC13 | Schedule for the Evaluation of Individual Quality of Life-Direct Weighting |
| European Organization for the Research and Treatment of Cancer Quality of Life Questionnaire-MY20 | Scoliosis Research Society 22r |
| European Organization for the Research and Treatment of Cancer Quality of Life Questionnaire-OV28 | Seattle Angina Questionnaire |
| European Organization for the Research and Treatment of Cancer Quality of Life Questionnaire-PR25 | Self-Assessment Manikin |
| EQ-5D | Self-Compassion Scale |
| EQ-6D | Self-Report Questionnaire on Adherence |
| Eysenck Personality Questionnaire | Sense of Coherence Scale |
| Family Environment Scale | Severe Respiratory Insufficiency questionnaire |
| Fatigue Impact Scale | Sexual Activity Questionnaire |
| Fatigue Severity Scale | Short Form-6D |
| Finnish Breast-Associated Symptoms questionnaire | Short Form-8 |
| Finnish Pain Questionnaire | Short Form-12 |
| Five Facet Mindfulness Scale | Short Form-20 |
| Florida Shock Anxiety Scale | Short Form-36 |
| Foot and Ankle Disability Index | Short Health Scale |
| Forgotten Joint Score 12 | Short Questionnaire to Assess Health Enhancing Physical Activity |
| Fragebogen zur Beurteiling der Behandling | Sino-nasal Outcome Test-22 |
| Functional Assessment of Cancer Therapy - Anemia | Social Difficulties Inventory |
| Functional Assessment of Cancer Therapy - Bladder | Social Network and Support Scale |
| Functional Assessment of Chronic Illness Therapy - Fatigue | Somatic Symptom Inventory |
| Functional Digestive Disorders Quality of Life Questionnaire | Somatoform Dissociation Questionnaire 20 |
| Functional Disability Questionnaire | Spine Pain Index |
| Functional Outcomes of Sleep Questionnaire | Sports Specific Scale |
| Gastrointestinal Symptom Rating Scale | St George`s Resporatory Questionnaire |
| General Health Questionnaire | State-Trait Anxiety Inventory |
| General Health Status | Stigma Scale for Chronic Illness |
| Geriatric Depression Scale | Stoma-Qol |
| German daily life ability scale Fertigkeitenskala Münster–Heidelberg | SWAL-QOL |
| German Social Support Questionnaire - Fragebogen zur sozialen Unterstützung | SWED-QUAL |
| German Social Support Questionnaire Fragebogen zur sozialen Unterstützung 14 | Symptom Checklist 27/90 |
| Glascow Outcome Scale Extended | Symptom Checklist-20 depression scale |
| Glaucoma Symptom Scale | Symptom Inventory ALS |
| Global Assessment of Functioning scale | Systemic Lupus Erythematosus Disease Activity Index 2000 |
| Global Percived Health | Tampa Scale of Kinesiophobia |
| Global Severity Index | Tegner Score |
| Groningen Activity Restriction Scale | Tinnitus Questionnaire by Goebel and Hiller |
| Hallam tinnitus questionnaire | Tinnitus triggers questionnaire |
| Hamilton Anxiety Scale | TNO-AZL Child Quality of Life Questionnaire |
| Hamilton Depression Scale | Toronto Alexithymia Scale |
| Harper Self-Perception Profile for Children and Adolescents | Traumatic Experiences Checklist |
| Harris Hip Score | Treatment Satisfaction Visual Analog Scale |
| Headache Impact Test | Tremor Rating Scale |
| Health Assessment Questionnaire | Tuebingen CD-25 |
| Health Assessment Questionnaire Disability Index | Type-D Scale |
| Health Complaints Scale | Unified Parkinson’s Disease Rating Scale |
| Health Education Literacy | University of California San Diego Shortness of Breath Questionnaire |
| Health of the Nation Outcome Scales | University of Washington Quality-of-Life Questionnaire |
| Health Utilities Index Mark | Uterine Fibroid Symptom and Health-related quality of life questionnaire |
| Hip Disability and Osteoarthritis Outcome Score | Utrecht Coping List |
| Hirschsprung disease/Anorectal malformation Quality of Life questionnaire | Vascular Quality of Life |
| HIV Symptom Distress Module | Ventral Hernia Pain Questionna |
| Homburg Varicose Vein Severity Score | Visual Function 14-item questionnaire |
| Hospital Anxiety and Depression Scale | Voice Handicap Index |
| Hyperhidrosis Disease Severity Scale | Ways of Coping Cancer version |
| Illness Perception Questionnaire | Ways of Coping Check List |
| Illness-specific Social Support Scale | Western Ontario & McMaster Universities Osteoarthritis Index |
| Impact On Family Scale | WHOQOL-BREF |
| Incontinence ImpactQuestionnaire | Work Competence Questionnaire |
| Infant and Toddler Quality of Life Questionnaire | Work Productivity and Activity Impairment |
| Inflammatory Bowel Disease Questionnaire | Work Productivity Impairment Questionnaire-Specific Health Problem |
| Institute for Personality and Ability Testing | Xerostomia Questionnaire |
| International Atomic Energy Agency scale | Yale-Brown Obsessive-Compulsive Scale |
| International Consultation on Incontinence Questionnaire | Yesavage Geriatric Depression Scale score |
| International Consultation on Incontinence Questionnaire Anal Incontinence Symptoms and Quality of Life Module | Youth Self-report |
| International Hip Outcome Tool 33 | Zarit Burden Interview |
| International Index of Erectile Dysfunction | Zung Self-Rating Depression Scale |
